# Special Populations in Alcoholics Anonymous

**Published:** 1998

**Authors:** J. Scott Tonigan, Gerard J. Connors, William R. Miller

**Affiliations:** J. Scott Tonigan, Ph.D., is deputy director of research and William R. Miller, Ph.D., is director of research of the Center on Alcoholism, Substance Abuse, and Addictions (CASAA), University of New Mexico, Albuquerque, New Mexico. Gerard J. Connors, Ph.D., is director of the Research Institute on Addictions, Buffalo, New York

**Keywords:** Alcoholics Anonymous, special populations, minority group, ethnic group, sociocultural norms, AODD (alcohol and other drug dependence) recovery, Hispanic, African American, white American, epidemiology, psychosocial treatment method, outpatient care, after-care, treatment outcome, AOD abstinence, comparative study, New Mexico, literature review

## Abstract

The vast majority of Alcoholics Anonymous (AA) members in the United States are white, and only a few studies have investigated the program’s effectiveness for ethnic minorities. Project MATCH, a multisite research study aimed at developing guidelines for assigning alcoholics to appropriate treatment approaches, also assessed AA effectiveness for minority clients. Some differences in AA attendance existed among white, African-American, and Hispanic Project MATCH participants who had received some inpatient treatment before entering the study, but not among participants who had not received inpatient treatment. Further analyses of white and Hispanic Project MATCH participants demonstrated that although Hispanic clients attended AA less frequently than white clients, their involvement with and commitment to AA was higher than among white clients. For both Hispanics and whites, AA involvement predicted increased abstinence.

Alcoholics Anonymous (AA) describes itself as a mutual-help program that is based on the attraction of its members to the program’s philosophy rather than on program promotion (Alcoholics Anonymous 1976). How attractive, however, is AA to ethnic minorities with alcohol-related problems? In other words, can a mutual-help program with strong Protestant roots that was started by white, middle-class Americans equally appeal to clients with diverse ethnic and cultural backgrounds? The spread of AA-based 12-step ideology and practices to 44 countries and the publication of the AA core literature in at least 8 languages would argue in the affirmative. Several factors may explain the growth and acceptance of AA across cultures (Makela 1993). For example, the 12-step philosophy is intentionally broad and open to divergent interpretations. This ideological flexibility permits its wide application across diverse cultures holding different beliefs and values. Furthermore, AA explicitly renounces political affiliations and shuns associations with other social movements. This “isolationism” has facilitated the introduction of AA into geopolitical areas that have traditionally discouraged the formation of grassroots social movements.

A slightly different question is whether minorities consider AA an attractive resource when the program is practiced within a larger dominant culture, such as that of the United States. In this situation, minority groups are asked to both adopt and modify the majority’s interpretations, values, and beliefs about what is most germane in 12-step ideology and practice. These conditions raise a series of related questions, such as the following: How do minority groups in the United States use AA? How, if at all, do practices among AA members vary because of ethnic and cultural differences? Finally, do ethnic and cultural factors influence the benefits associated with AA attendance and involvement? This article addresses those questions based on findings obtained from epidemiological studies, the Project MATCH treatment study, and analyses of two samples of Hispanic clients with alcohol problems recruited in Albuquerque, New Mexico.

## Findings From Epidemiological Research

Epidemiological analyses in the general population of the United States indicate that AA is well known among Hispanics and African-Americans. Moreover, a vast majority of the people in those ethnic groups generally would recommend AA affiliation for alcohol-related problems (Caetano 1993). Prevalence estimates vary considerably, however, on the extent to which African-American and Hispanic clients actually select AA as a resource. For example, Caetano (1993) suggested that the proportion of people among the general population who were likely to attend AA was greater among Hispanics (12 percent) than among African-Americans (5 percent) or whites (5 percent). In contrast, Humphreys and Moos (1996), in a sample of clients with primary alcohol dependence, found no ethnic preferences as to whether the clients selected formal outpatient treatment or AA. Finally, the 1996 AA membership survey (Alcoholics Anonymous 1997) indicated that about 4 percent of its members were Hispanic and 5 percent were African-American.

## Findings From Project Match

Project MATCH is a multisite research study aimed at developing practical guidelines for assigning patients with alcohol problems to appropriate treatment based on patients’ characteristics (Project MATCH Research Group 1993, 1997). Study participants were recruited at 10 locations throughout the country and were randomly assigned to one of three psychosocial therapies: (1) cognitive behavioral therapy, (2) motivational enhancement therapy, or (3) 12-step facilitation therapy. Cognitive behavioral therapy consisted of skills training to achieve the treatment goals—that is, teaching clients the skills necessary to cope with situations (e.g., stress or social occasions at which alcohol is served) that might induce drinking. Additional emphasis was placed on skills thought necessary to avoid a full relapse should drinking occur. Motivational enhancement therapy, in contrast, supportively encouraged the clients to take responsibility for changes in their behavior. This approach focused on enhancing the client’s self-efficacy and mobilization of resources to promote and sustain change. Finally, 12-step facilitation therapy guided the clients through the first five steps of the AA program and actively promoted affiliation with AA. All three therapy approaches were manual guided, and each participating therapist administered only one type of therapy.

All clients were offered 12 weeks of the assigned therapy on an outpatient basis. During that time, AA attendance was neither promoted nor discouraged in the cognitive-behavioral and motivational-enhancement therapy approaches. After the treatment period, the clients were followed at 3-month intervals for 12 months. At each followup, their AA attendance and AA involvement^1^ were determined. An initial analysis has suggested that more than 70 percent of the entire Project MATCH sample elected at least minimal AA attendance and that more than 30 percent of the sample attended AA throughout the 12 months of followup (Tonigan et al. in press).

The study included two groups of participants: (1) the aftercare sample, who had already completed at least 7 days of residential treatment before being recruited to the study, and (2) the outpatient sample, who had received no residential treatment (for a more detailed description of the samples, see Project MATCH Research Group 1997). Most clients in both samples fulfilled the diagnosis of alcohol dependence and reported no other current drug dependence (aside from marijuana use).

Because Project MATCH also included minority clients, the study’s findings can be used to examine minority participation in AA after formal treatment. In fact, Project MATCH offers a unique perspective on AA participation among different ethnic groups for two reasons. First, the study included both clients who did and clients who did not receive residential treatment (i.e., the aftercare and outpatient samples). Second, the measures with which AA attendance and involvement were determined had strong reliability and were corroborated by independent sources (Tonigan et al. 1996, 1997, in press).

**Figure 1:**
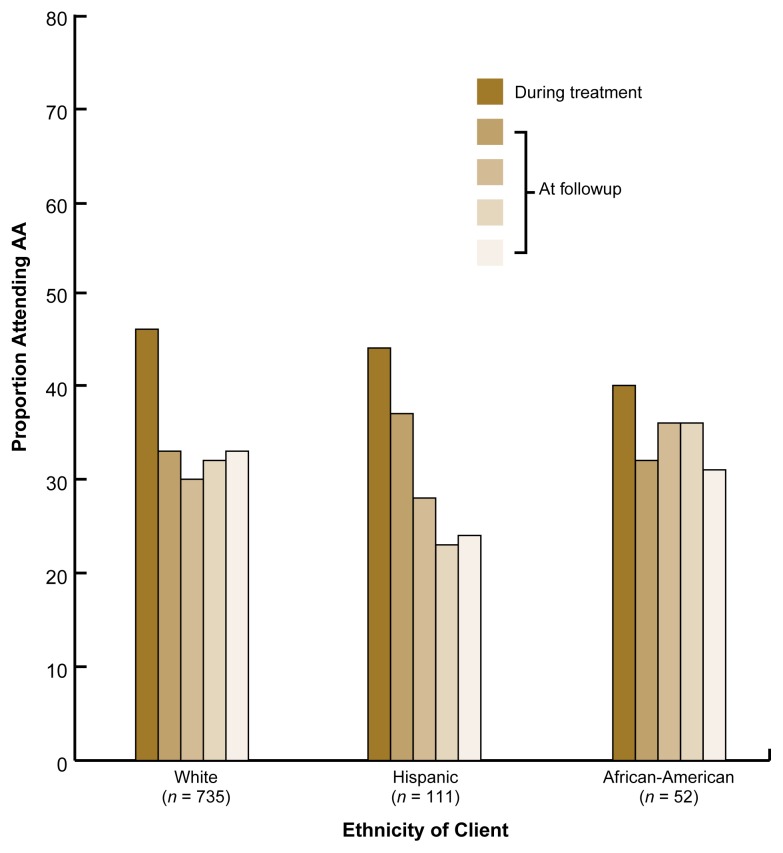
Proportion of clients of different ethnicities in the Project MATCH outpatient sample who reported Alcoholics Anonymous (AA) attendance during and after treatment. The clients received 12 weeks of psychosocial therapy (i.e., cognitive-behavioral therapy, motivational-enhancement therapy, or 12-step-facilitation therapy) without previous residential treatment. The clients’ AA attendance was assessed during the 12-week treatment period as well as during four consecutive 3-month followups (shown as increasingly lighter shades of gold over time). No significant differences in the rates of AA attendance existed among ethnic groups.

The proportions of clients of various ethnicities who attended any AA meetings during treatment and during the four consecutive 3-month followup periods differed between the outpatient and aftercare samples. In the outpatient sample, the client’s ethnicity (i.e., white, Hispanic, or African-American) did not predict AA attendance at any followup point after controlling for the psychosocial treatment the clients had received (figure 1): Relatively equivalent proportions of each ethnic group attended AA. These findings are similar to those reported in single-group studies in which clients were not randomly assigned to different treatments (e.g., Humphreys et al. 1994; Humphreys and Moos 1996). Furthermore, no differences in AA attendance existed among ethnic groups in the outpatient sample after a long-term followup (i.e., after 3 years).

**Figure 2:**
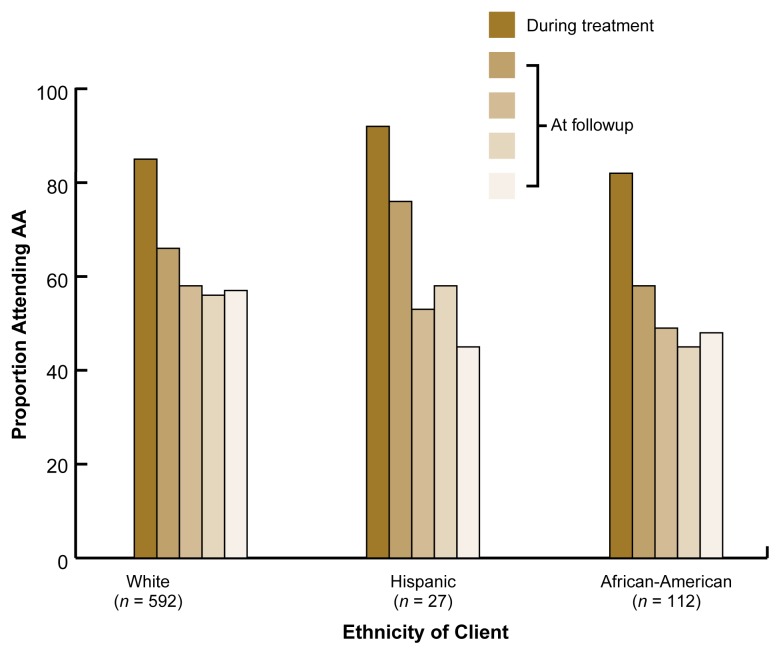
Proportion of clients of different ethnicities in the Project MATCH aftercare sample who reported Alcoholics Anonymous (AA) attendance during and after treatment. The clients had completed at least 7 days of residential treatment before receiving 12 weeks of psychosocial therapy (i.e., cognitive-behavioral therapy, motivational-enhancement therapy, or 12-step-facilitation therapy). The clients’ AA attendance was assessed during the 12-week treatment period as well as during four consecutive 3-month followups (shown as increasingly lighter shades of gold over time). Some differences in the rates of AA attendance existed among ethnic groups.

In the Project MATCH aftercare sample, the proportion of clients who attended any AA meetings generally was higher than in the outpatient sample (figure 2). In addition, some ethnic differences existed in AA attendance during the 12-month followup period. Proportionally fewer African-American than Hispanic or white clients reported AA exposure during the first 6 months after treatment. In addition, the difference between African-Americans and whites became statistically significant during the last 3 months of the 12-month followup period. Other studies, however, have reported that African-Americans were as likely to attend AA after residential treatment as were whites and that AA attendance was beneficial^2^ to African-Americans (Humphreys et al. 1994). No difference in AA exposure existed between Hispanics and whites in the aftercare sample during the first 9 months after treatment. During the last 3 months of the followup period, however, significantly fewer Hispanics than whites reported any AA exposure, and no significant differences in AA exposure existed between Hispanic and African-American clients.

## Studies Comparing Hispanic and White Clients

The frequency of AA attendance and the associated benefits for Hispanic and white clients who had received formal treatment were investigated in more detail in a long-term (i.e., longitudinal) study of Hispanic and non-Hispanic clients in Albuquerque, New Mexico, and in the Hispanic and white clients recruited in Albuquerque for the Project MATCH study. The first of these two studies examined factors associated with relapse (Miller et al. 1996). In this study, Hispanic clients attended AA significantly less frequently than did non-Hispanic clients during the 6 months after study recruitment. Conversely, the Hispanic clients reported attending a significantly higher number of formal outpatient treatment sessions than did the non-Hispanic clients (Arroyo et al. 1998). Regardless of ethnic group membership, however, AA attendance was associated with significantly less intense drinking when drinking did occur and with significantly lower total alcohol consumption.

Similar attendance patterns also were seen in the Project MATCH study, in which the Albuquerque clinical site recruited the majority of the Hispanic clients (i.e., 100 out of 111) in the outpatient sample. Again, Hispanic clients had significantly lower rates of AA attendance than did white clients during the early followup periods. This difference decreased, however, at the 12-month and 3-year followups. It is noteworthy that in contrast to other cities, AA meetings held in Spanish are readily available in Albuquerque. Thus, it is unlikely that lower rates of AA attendance among Hispanic clients in the two studies were a result of language barriers. In fact, the AA attendance estimates obtained in these studies may be even higher than what may be expected in less culturally sensitive regions.

**Figure 3:**
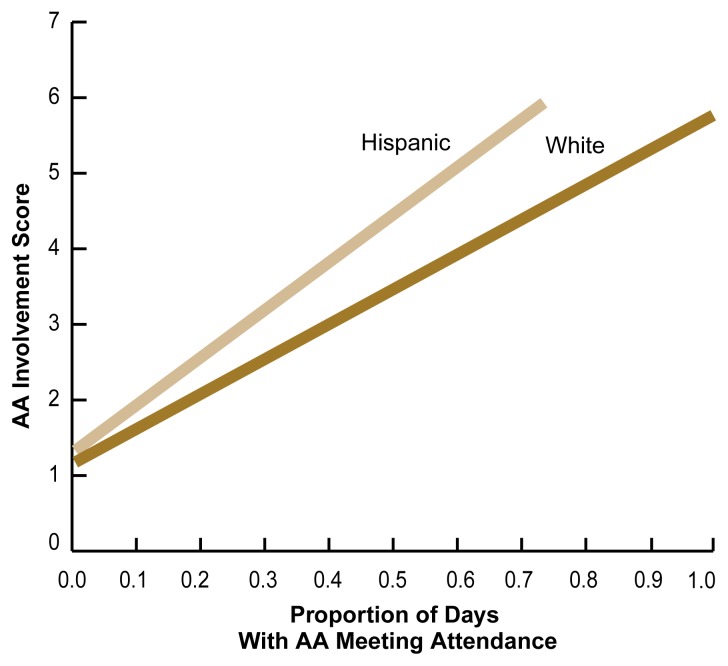
Relationship between Alcoholics Anonymous (AA) attendance and AA involvement in Hispanic and white clients recruited for the Project MATCH study in Albuquerque, New Mexico. AA attendance and involvement were determined 6 months after the clients had completed a 12-week course of psychosocial therapy (i.e., cognitive-behavioral therapy, motivational-enhancement therapy, or 12-step-facilitation therapy). AA attendance was expressed as the proportion of days on which the clients attended an AA meeting. The AA involvement score included such measures as to what extent the clients practiced each of the 12 steps, had or were a sponsor, or celebrated AA birthdays. Hispanic clients reported a higher level of AA involvement despite lower AA attendance. SOURCE: Tonigan et al. 1996.

Because AA involvement and commitment to AA-related principles and practices better predict a successful outcome than does mere AA attendance (Montgomery et al. 1995), one can also ask whether Hispanic clients who elect to attend AA become as engaged in AA-related activities as do their white counterparts. To address this question, Tonigan and colleagues (1996) investigated the relationship between AA attendance and AA involvement at 6 months after treatment for both Hispanics and non-Hispanics recruited for the Project MATCH study. Some of the measures of AA involvement in this study included the extent to which AA participants practiced each of the 12 steps, had or were a sponsor, and celebrated AA birthdays. The study found that compared with whites, Hispanic clients reported higher levels of commitment to AA-related practices despite lower AA attendance (figure 3). These findings suggest that for those Hispanics who elect to attend AA, the program’s practices may be readily acceptable and easily adopted.

Current evidence suggests that AA attendance after treatment is modestly related to abstinence (Emrick et al. 1993). The influences of ethnicity and the type of formal treatment received on outcome (e.g., abstinence), however, have not been evaluated systematically. The two studies among Hispanics described earlier in this section statistically controlled for the type of treatment the clients had received. These analyses detected no differential benefit associated with AA attendance based on client ethnicity. For both Hispanics and whites, greater frequency of AA attendance was associated with an increase in the percentage of abstinent days during followup. As in previous studies, AA involvement predicted a positive outcome more strongly than did AA attendance for the Albuquerque Project MATCH outpatient sample, but this relationship did not depend on the clients’ ethnicity. It is not known, however, whether ethnicity mediates potential benefits of AA in other domains, such as increased purpose in life, reduced depression, and improved legal or employment status. This question certainly warrants additional research.

## Preliminary Conclusions

The findings described in this article allow several tentative conclusions. First, the modest positive association between AA attendance and abstinence that has been reported previously (Emrick et al. 1993) appears to apply to all AA members regardless of their ethnic backgrounds. The studies conducted to date provide no evidence that the drinking status of people who elect to attend AA is affected by their ethnicity. These conclusions, however, are based on studies involving preselected participants who had sought formal treatment. Consequently, it remains unknown whether the benefits associated with AA attendance apply equally to people with various ethnic backgrounds who do not seek formal treatment.

Second, analyses of the attractiveness of AA to minority groups have yielded complex findings that defy simple interpretations. On the one hand, survey research has indicated that in contrast to whites, fewer Hispanics and African-Americans attend AA than attend formal treatment. On the other hand, two clinical trials evaluating outpatient treatment reported inconsistent findings about the likelihood of Hispanic clients attending AA at the same rate as did white clients. It is important to note, however, that the study by Arroyo and colleagues (1998), which reported less AA utilization by Hispanic clients, only had a relatively short followup period of 6 months. It is unclear whether the ethnic differences reported in that study would have persisted over longer followup periods. The second study—the Project MATCH aftercare sample—found the reverse situation: In that study, proportionally fewer Hispanic and African-American clients attended AA during late followup periods, but no differences from whites existed during early followup. These apparently contradictory findings indicate that global questions regarding ethnic rates of AA utilization should be discarded in favor of more specifically focused questions that consider contextual factors, such as the time since the cessation of treatment.

Finally, in the studies described in this article, Hispanic clients reported greater gains in AA involvement while attending fewer AA meetings compared with white clients. The assessment of minority utilization of AA therefore should go beyond simple measurements of the frequency of AA attendance, because such measurements might underestimate the influence of AA on the recovery efforts of minority clients.

## Footnotes

1 AA involvement refers to the client’s commitment to AA-related principles and practices and therefore differs from mere AA attendance. One study has suggested that AA involvement is a better predictor of outcome than AA attendance (Montgomery et al. 1995).

2 Beneficial outcomes included increased employment, decreased alcohol and other drug use, and improved legal status.
